# Physiological Regulation of Isocitrate Dehydrogenase and the Role of 2-Oxoglutarate in *Prochlorococcus* sp. Strain PCC 9511

**DOI:** 10.1371/journal.pone.0103380

**Published:** 2014-07-25

**Authors:** María Agustina Domínguez-Martín, Antonio López-Lozano, Jesús Diez, Guadalupe Gómez-Baena, Oriol Alberto Rangel-Zúñiga, José Manuel García-Fernández

**Affiliations:** Departamento de Bioquímica y Biología Molecular, Universidad de Córdoba, Córdoba, Spain; University of Freiburg, Germany

## Abstract

The enzyme isocitrate dehydrogenase (ICDH; EC 1.1.1.42) catalyzes the oxidative decarboxylation of isocitrate, to produce 2-oxoglutarate. The incompleteness of the tricarboxylic acids cycle in marine cyanobacteria confers a special importance to isocitrate dehydrogenase in the C/N balance, since 2-oxoglutarate can only be metabolized through the glutamine synthetase/glutamate synthase pathway. The physiological regulation of isocitrate dehydrogenase was studied in cultures of *Prochlorococcus* sp. strain PCC 9511, by measuring enzyme activity and concentration using the NADPH production assay and Western blotting, respectively. The enzyme activity showed little changes under nitrogen or phosphorus starvation, or upon addition of the inhibitors DCMU, DBMIB and MSX. Azaserine, an inhibitor of glutamate synthase, induced clear increases in the isocitrate dehydrogenase activity and *icd* gene expression after 24 h, and also in the 2-oxoglutarate concentration. Iron starvation had the most significant effect, inducing a complete loss of isocitrate dehydrogenase activity, possibly mediated by a process of oxidative inactivation, while its concentration was unaffected. Our results suggest that isocitrate dehydrogenase responds to changes in the intracellular concentration of 2-oxoglutarate and to the redox status of the cells in *Prochlorococcus*.

## Introduction

The marine cyanobacterium *Prochlorococcus* [1], [2] has become an important model marine microbe for ecological studies since its discovery [3], [4], because of its abundance and significant contribution to global primary production [5]. Thirteen *Prochlorococcus* genomes [6]–[9], representative of the different ecotypes [10], [11], have been sequenced to date. However, an important fraction of the genomic information has been obtained by comparison with other organisms, while there are not many physiological studies carried out in *Prochlorococcus* [12]–[16], due to the problematic culturing of this microorganism [17]. Consequently, there is a clear need for *in vivo* studies addressing the physiology of *Prochlorococcus* [18], [19], in order to further understand the underpinnings of differences among ecotypes, which might illuminate the reasons explaining the tremendous ecological success of this organism. This led our team to study the metabolism of nitrogen assimilation in several *Prochlorococcus* strains [17], [20]–[25].

Isocitrate dehydrogenase (ICDH, EC 1.1.1.42) appeared as an ideal candidate enzyme to start physiological studies on the C/N interface in *Prochlorococcus*, due to its position at the branching point between carbon and nitrogen metabolic pathways. This enzyme catalyzes the oxidative decarboxylation of isocitrate, producing 2-oxoglutarate (2-OG) and reducing NADP^+^ to NADPH. For many years, it was believed that cyanobacteria possessed an incomplete tricarboxilic acids (TCA) cycle [26], [27] (lacking 2-OG dehydrogenase, among other enzymes). However, a recent study [28] demonstrated the occurrence in most cyanobacteria of two enzymes (2-oxoglutarate decarboxylase and succinic semialdehyde dehydrogenase), which actually close the TCA cycle, allowing the transformation of 2-OG to succinate. Interestingly, the same study showed that marine *Synechococcus* and *Prochlorococcus* strains do lack both enzymes. Consequently these groups seem to be the only cyanobacteria which actually possess an incomplete TCA cycle. Beneficial interactions between *Prochlorococcus* and coexistent heterotrophic bacteria have been demonstrated, mediated in some cases by diffusible compounds [29]–[31]. Therefore one could hypothesize that some of the missing metabolites of the TCA cycle could be taken up by *Prochlorococcus* from the environment in order to close the cycle, in a manner similar to the uptake of vitamin B_12_ observed in marine plankton [32]. Although transporters of organic acids appear in the *Prochlorococcus* genomes (for instance, the gene PMN2A_1447 in *Prochlorococcus marinus* sp. NATL2A, annotated as di/tricarboxilate transporter), this possibility seems improbable, due to the scarcity of organic molecules in the ocean and to the high concentration of heterotrophic bacteria, which would outcompete *Prochlorococcus* in scavenging such compounds.

**Figure 1:**
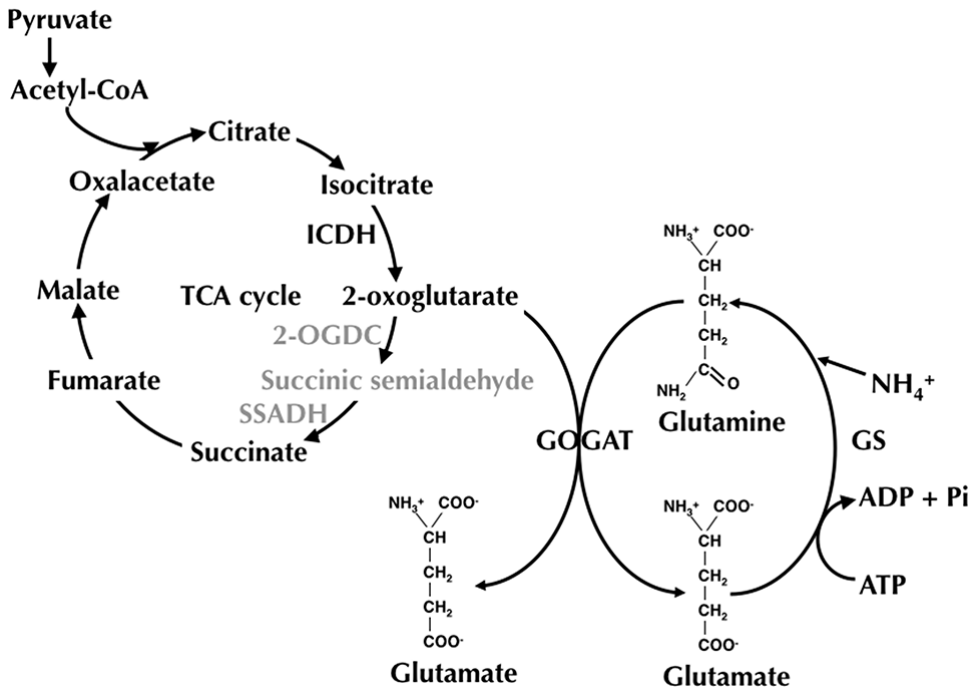
Outline of the pathways for 2-OG metabolism in cyanobacteria. 2-OG, produced from isocitrate in the TCA cycle, can be used by all cyanobacteria as backbone to incorporate ammonium, through the GS/GOGAT pathway. Alternatively, the majority of cyanobacteria can transform 2-OG to succinic semialdehyde, and later to succinate, through reactions catalyzed by 2-OG decarboxylase (2-OGDC) and succinic semialdehyde dehydrogenase (SSADH), respectively [28]. These reactions (shown in grey) are missing in marine *Synechococcus* and *Prochlorococcus* strains.

This means a metabolic background entirely different with respect to the rest of cyanobacteria: while 2-OG can be metabolized through two different pathways in the majority of cyanobacteria (either the TCA cycle, or the glutamine synthetase-glutamate synthase (GS-GOGAT) pathway), in *Prochlorococcus* and marine *Synechococcus*, it can only be metabolized through GS-GOGAT (Fig. 1). Furthermore, glutamate dehydrogenase (whose aminating reaction allows the production of glutamate from ammonium and 2-OG in other bacteria) is either lacking [7], [8], [33] or acting in the deaminating direction in marine cyanobacteria [34], and therefore does not allow the use of 2-OG to produce glutamate. In this context, the interest on the regulation of ICDH in *Prochlorococcus* is enhanced, since the 2-OG produced by ICDH cannot be further oxidized, and thus can only be used as the carbon skeleton through the GS-GOGAT cycle for ammonium assimilation.

ICDH has been purified in five cyanobacterial strains (*Anacystis nidulans* [35], *Synechocystis* sp. PCC 6803 [36], *Anabaena* sp. PCC 7120 [37], *Phormidium laminosum* [38] and *Microcystis aeruginosa* sp. PCC 7806 [39]), the latter expressed in *E. coli*, being composed of two identical subunits of 55 kDa. Besides, the gene *icd*, encoding ICDH, has been cloned from *Synechocystis* sp. PCC 6803 [40], *Anabaena* sp. PCC 7120 [37], and *Microcystis aeruginosa* PCC 7806 [39], and it is found in all cyanobacterial genomes thus far available (including all the *Prochlorococcus* and *Synechococcus* strains). This fact, together with the impossibility to segregate *icd* mutants observed in *Synechocystis* sp. PCC 6803 and *Anabaena* sp. PCC 7120 [37], [40], strongly suggests that *icd* is an essential gene for cyanobacteria.

Very little is known about the physiological regulation of ICDH in cyanobacteria. The few published studies were focused on the effect of nitrogen sources or nitrogen starvation [36], [38], [40]–[42]. While ICDH activity did not significantly change when cells were growing on different nitrogen sources [36], [43], the enzyme activity [38], [40]–[42], enzyme concentration [41] and *icd* expression [40] increased under nitrogen starvation.

2-OG is a product of the ICDH reaction, and the main molecule utilized by cyanobacteria to sense the balance between the C and N metabolisms [44]–[46]. Palinska and coworkers studied the PII protein in *Prochlorococcus* sp. PCC 9511, proposing that it might act as a 2-OG sensor, and that 2-OG may play a role in regulation of inorganic carbon acquisition through the 2-OG-PII complex [14]. However, no specific study has been carried out thus far addressing the role of 2-OG in marine cyanobacteria. Taking into account the simplification of the regulatory networks observed in *Prochlorococcus* [7], [8], [22], it seems plausible to expect changes in the mechanisms for perception of the C/N balance, and consequently we decided to analyze in detail the regulation of ICDH, the enzyme catalyzing the conversion of isocitrate to 2-OG. We were particularly interested in the effects of azaserine, a specific inhibitor of GOGAT [47], [48]. This inhibition presumably provokes a significant increase in the concentration of 2-OG, which can neither be incorporated into glutamate by means of the GOGAT, nor metabolized through the TCA cycle because of its incompleteness in *Prochlorococcus* (Fig. 1). Previous studies on GS regulation in *Prochlorococcus* showed that azaserine addition has a strong effect on GS activity, inducing a large increase in both the activity and the concentration of the enzyme in *Prochlorococcus* [17].

In this work, we have performed an initial characterization of ICDH from *Prochlorococcus* sp. PCC 9511; we addressed its behaviour *in vivo* under different physiological conditions, related with key changes that could affect *Prochlorococcus* in the ocean, measuring enzyme activity by a spectrophotometric assay and enzyme concentration by Western blotting. In the experimens addressing the effect of azaserine, we also quantified *icd* expression by qRT-PCR. Besides, we studied the effect of metal-catalyzed oxidative systems on this enzyme, as well as the effect of 2-OG on its regulation.

## Materials and Methods

### Chemicals

All chemicals were of reagent grade, obtained from *Merck* or *Sigma*.

### *Prochlorococcus* strains and growth conditions

*Prochlorococcus marinus sp*. strain PCC 9511 (HL-adapted, axenic) was routinely cultured in Polycarbonate Nalgene flasks (10 L) using PCR-S11 medium as described [49]. The seawater used as basis for this medium was kindly provided by the Instituto Español de Oceanografía (Spain). Cells were grown in a culture room set at 24°C under continuous blue irradiance (40 µE m^2^s^−1^). Cells were collected during the exponential phase of growth. Growth was determined by measuring the absorbance of cultures at 674 nm. DCMU (diuron, 3-(3–4 dichlorophenyl)-1,1-dimethylurea) and DBMIB (2,5-dibromo-3-methyl-6-isopropyl-p-benzoquinone) were dissolved and added to cultures as previously described [17].

### Cell collection

Cells were centrifuged at 30,100 *g* for 5 min in an *Avanti J-25 Beckman* centrifuge equipped with a *JA-14* rotor. When large volumes were required (>10 L), centrifugations were performed at 18,600 *g* for 8 min in a *JLA-10.500 Beckman* rotor. After pouring most of the supernatant and carefully pipetting out the remaining medium, the pellet was directly resuspended in 2 mL of cold 50 mM Tris-HCl pH 7.5 buffer per liter of culture, and immediately frozen at −80°C until used. When cell extracts were prepared for Western blotting or to determine 2-OG, the concentration was increased 4-fold (cells from 1 L of culture resuspended in 0.5 mL of buffer).

In nutrient starvation experiments, cultures were centrifuged as described above. The pellets were washed with nutrient-free PCR-S11 medium, and finally diluted with the same original volume of media, by using standard PCR-S11 for controls, or media lacking either nitrogen, phosphorus, or iron. Two aliquots were prepared from each culture (control and nutrient-depleted), which were subjected to standard conditions of light and temperature. Samples were taken at the indicated time following the protocol described above.

### Cell extracts

For enzymatic assays or Western blotting, the cell suspensions were broken in a French pressure cell (*SLM/Aminco* model *FA-079*) at 16,000 psi; the obtained extracts were centrifuged for 10 min at 16,900 *g* and 4°C. To determine 2-OG concentration cell extracts were obtained by centrifuging the thawed extracts for 10 min at 16,900 *g* and 4°C.

### Enzymatic assays

ICDH assay was based on that described by Muro-Pastor and Florencio [36], with modifications. The reaction mixture contained 840 µL of 50 mM Tris pH 7.5, 20 µL of 100 mM MnSO_4_, 20 µL of 10 mM NADP^+^, 20 µL of 100 mM D, L-isocitrate and 100 µL of cell extract. Isocitrate was added last to start the reaction. NADPH production was monitorized by determining absorbance at 340 nm for 10 min, in quartz cuvettes thermostatized at 40°C. The slope of the curve between 6 and 10 min was used to calculate the ICDH activity. One unit of activity is the amount of enzyme that produces 1 µmol of NADPH per minute. ICDH activity is expressed as mU of activity per mg of total protein. The determined values of ICDH activity shown in the different experiments were well above the detection limits of this assay.

Protein concentration was determined using the *Bio-Rad Protein Assay kit*, based on the method described by Bradford [50].

### Inactivation assays

For the inactivation of ICDH by the Fe^3+^/ascorbate system [21], [25], incubations were carried out at 4°C in a solution containing 50 mM Tris-HCl buffer pH 7.6, 0.2 mM FeCl_3_ and variable concentrations of ascorbate (1, 5 or 10 mM). The ascorbate solution was prepared as follows: 100 µL of 20 mM dithiothreitol were added to 9 mL of 100 mM ascorbate, in order to keep ascorbate under reduced conditions; after carefully neutralizing this solution with 1 mM NaOH, the volume was adjusted to 10 mL.

The inactivation reactions were initiated by the addition of ascorbate. Distilled water further purified with a *Millipore MilliQ* system was used in the preparation of the inactivation reaction mixtures to avoid the effect of metal traces.

### Determination of the intracellular 2-OG concentration

An enzymatic method based on the oxidation of NADPH in the reaction catalyzed by the glutamate dehydrogenase was used [51]. This enzyme catalyzes the production of glutamate from ammonium and 2-OG, using NADPH as reducing power with a stechiometry of 1∶1. The reaction was optimized for *Prochlorococcus* samples. The reaction mixture contained: 85 mM Tris-HCl pH 8.0, 0.2 mM NADPH, 5 µg (ca. 0.15 UI) of glutamate dehydrogenase enzyme (*Fluka*), 100 mM NH_4_Cl and 200 µL of cell extract from *Prochlorococcus* (total volume of enzymatic mixture was 1 mL). The NADPH consumption was monitorized by measuring the absorbance at 340 nm for 10 min, in quartz cuvettes thermostatized at 35°C.

### Detection of isocitrate dehydrogenase by Western blotting

Crude extracts from *Prochlorococcus* were prepared as described above. 15 µg of protein were loaded in each lane, subjected to SDS-PAGE and transferred to nitrocellulose membrane. After transfer the membrane was stained with Ponceau S (0.2%) in 5% acetic acid to check for equivalent protein loading and transfer efficiency, and then treated as follows: washing for 15 min with TBS-T (20 mM Tris-HCl pH 7.4, 150 mM NaCl and 0.1% Tween 20); blocking with TBS-T containing 1% bovine seroalbumin for 2 h and 3-fold washing for 15 min with TBS-T buffer. Overnight incubation with primary antibody (anti-isocitrate dehydrogenase from *Synechocystis* sp. PCC 6803, kindly provided by Dr. M. I. Muro-Pastor and Prof. F. J. Florencio) diluted 1∶4000 (v/v) in TBS-T 1% bovine seroalbumin, at 4°C with gentle shaking. Washing 3-fold for 15 min with TBS-T buffer. Incubation with secondary antibody (anti-immunoglobulin from rabbit, linked with peroxidase, *Sigma*) diluted 1∶2000 (v/v) in TBS-T for 30 min at room temperature with gentle shaking. Washing 3-fold for 15 min with TBS-T Buffer. The immunoreacting material was detected by using the *ECL Plus Western Blotting Detection System* (*General Electric Healthcare*), according to the manufacturer instructions. Chemiluminescent signal was detected using a *LAS-3000* camera (*Fujifilm*). The densitometric quantification of the Western blotting bands was performed by using the *Quantity One* software from *Bio-Rad*.

### RNA isolation

RNA was isolated from 500 mL cultures aliquots subjected to several conditions. Cells were harvested by centrifugation at 26,000 *g* for 8 min at 4°C. After pouring most of the supernatant and carefully pipetting out the remaining medium, the pellet was directly resuspended in 250 µL of cold buffer containing 10 mM sodium acetate pH 4.5, 200 mM sucrose and 5 mM EDTA, and stored frozen at −80°C until used. Total RNA was extracted using *TRIsure RNA Isolation Reagent* (*Bioline*) as recommended by the manufacters, except that an additional LiCl precipitation step was included at the end of the procedure to improve the RNA quality. RNA was treated with RNAse-free DNAseI (*Ambion*) following the manufacturer instructions, and the absence of contaminating genomic DNA was assessed using a PCR test.

### Real-time quantitative RT-PCR analysis of gene expression

The synthesis of the cDNA by the reverse transcriptase (RT) reaction from the RNA samples, was carried out using the *iScript cDNA Synthesis* kit from *Quanta* as recommended by the manufacturers. For a 20 µL of total volume reaction, 1 µg of RNA were reverse transcribed. Specific primers to amplify fragments of the *icd* gene (gene ID 1726275) from the *Prochlorococcus* strain MED4 (which is genetically identical to PCC 9511) were designed using the software Oligo 4.05 (*Molecular Biology Insights, Inc*.), on the basis of the corresponding *Prochlorococcus* MED4 genome [8]. During the optimization of qRT-PCR reactions, products were checked for single amplification of DNA fragments of the expected size by agarose gel electrophoresis. The sequences of the primers used were:

FG: 5′AGACTGCATTACGGAAAGAGAAAGC 3′ and

RGH: 5′CAGCAGCAGCATCAGAAACATAATC 3′.

Real time quantitative PCR reactions were performed in triplicate. The reaction mixtures contained 1× concentration of *SsoFast EvaGreen Supermix* from *Bio-Rad*, 0.128–0.384 µM forward and reverse primers (depending on the efficiency calculations) and the corresponding cDNA. The efficiency of the reactions was calculated and optimizated following the method described previously [52].

An *iCycler IQ* multicolor real time PCR detection system from *Bio-Rad* was used for quantitative detection of amplified PCR products using the following thermal cycling conditions: 95°C for 2 min, and 50 cycles of 95°C for 15 s, followed by 58°C for 30 s and 72°C for 30 s. At the end, reactions were checked to discard false amplifications by verifying the melting point of PCR products, determining the fluorescence between 65–100°C, with increases of 0.5°C, measured each 10 s.

Measurements were carried out in triplicate from at least three independent biological samples subjected to identical culture conditions. The relative change in gene expression was endogenously normalized to that of the gene *rnpB* (FE: 5′ACAGAAACATACCGCCTAAT3′ and RE: 5′ACCTAGCCAACACTTCTCAA 3′), encoding RNase P, calculated using the 2^−ΔΔCt^ method [52]. No change of the expression of *rnpB* was confirmed under our experimental conditions.

### *Prochlorococcus* genomic sequences

Cyanobacterial genomic data were obtained from the Joint Genome Institute (http://genome.jgi-psf.org) and Cyorf (http://cyano.genome.ad.jp/).

### Statistical analysis

Experiments were carried out at least with three independent biological samples. The results are shown with error bars corresponding to the standard deviation. Significance of data was assessed by using the Student's T test, and indicated in figures with asterisks: * means *p*≤0.05 ; ** means *p*≤0.01.

## Results and Discussion

### Characterization of ICDH activity from *Prochlorococcus* sp. PCC 9511

In order to detect ICDH activity in *Prochlorococcus* samples, we utilized a method previously described [36], introducing the necessary modifications to optimize its performance. The assay of ICDH activity was characterized by preparing different mixtures, each of them excluding one of the components of the reaction. In the absence of crude extract, isocitrate or NADP^+^, no activity was found. The samples without added manganese showed a 25% of the control activity (complete mixture), while those where manganese was replaced by magnesium had roughly half of the control activity, indicating a preference of ICDH for the manganese ion. Similar results have been observed in other bacteria, including cyanobacteria [36], [38], [39], [53], [54]. In a series of assays at different temperatures, we observed that ICDH activity increased continuously from 30 to 55°C, but at the same time it became less stable: preincubation of the cell extracts at different temperatures showed that the activity decreased sharply above 35°C. Similar results have been described in *M. aeruginosa* PCC 7806 [39]. Therefore, the standard assay temperature was set up at 40°C. There was an initial lag in all cases, regardless of the substrate addition order or the composition of the reaction mixture; this has been described as well in *Synechocystis* sp. PCC 6803 [36], and is probably due to the time required for the cation-isocitrate complex formation.

The stability of ICDH in crude extracts was followed by time course experiments; we observed the activity to be stable for at least 24 h. Addition of different stabilizing reagents (glycerol), protease inhibitors (EDTA, PMSF) or reducers (DTT) did not affect the stability of the enzyme. The apparent optimal pH of the ICDH from *Prochlorococcus* sp. PCC 9511 was found to be ca. 9.0–9.5. This value is close to those described for other NADP-ICDH [35]–[38], [55]–[57], with the exception of *M. aeruginosa*, which had an optimum pH of 7.5 [39], although it has to be taken into account that this was a recombinant enzyme.

Oxalomalate has been reported to be a powerful inhibitor of NADP-dependent ICDH, both in prokaryotes and eukaryotes [58]. We found no significant inhibitory effect on ICDH activity from *Prochlorococcus* (oxalomalate concentrations from 0 to 16 mM) with respect to mouse liver extracts, where a clear inhibition was observed when oxalomalate was added (not shown).

In summary, the characterization of ICDH in *Prochlorococcus* sp. strain PCC 9511 did not show any significant difference with respect to this enzyme in other organisms, with the exception of the lack of effect observed for oxalomalate.

### Effect of key nutrients starvation

In our previous studies on the regulation of GS, we observed that several conditions known to cause strong effects on its regulation in other cyanobacteria lacked such effect in *Prochlorococcus* [17], [24]; thus we were interested to check whether this might be due to a general streamlining on regulatory networks or to some specific response of glutamine synthetase. Therefore, we decided to study the effect of the absence of three key elements in the ocean, namely nitrogen, phosphorus and iron, on ICDH activity and compare the results with those obtained on glutamine synthetase.

**Figure 2:**
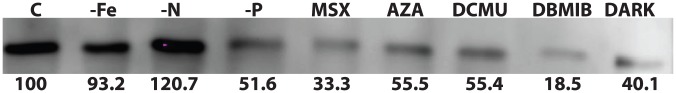
ICDH Western blotting of cell extracts from *Prochlorococcus* sp. strain PCC 9511 cultures subjected to different conditions. Cultures were subjected to the indicated conditions for 24(except in the case of iron starvation, which were starved for 8 h). C, control; -Fe, iron starvation; -N, nitrogen starvation; -P, phosphorus starvation; MSX, 100 µM methionine sulfoximine; AZA, 100 µM azaserine. The quantification of bands is shown below the picture, assigning an arbitrary value of 100 to the control conditions.

**Figure 3:**
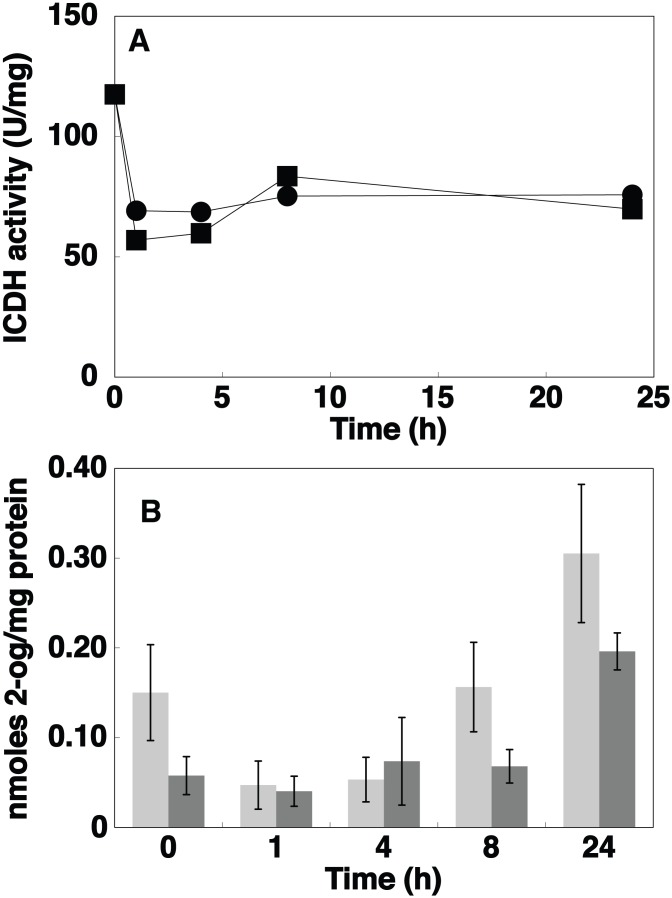
Effect of nitrogen starvation. A, Time course of ICDH activity in *Prochlorococcus* sp. strain PCC 9511 cultures (squares, control cells; circles, N-starved cells). B. Time course of 2-OG concentration (light grey, control cells; dark grey, N-starved cells). Values are the average of three independent biological replicates, each of them measured in triplicate. Error bars correspond to the standard deviation.

Ammonium is the preferred nitrogen source by cyanobacteria [59], [60], and one of the sources readily assimilated by all *Prochlorococcus* strains thus far studied. We studied the effect of nitrogen starvation for 24 h on ICDH activity and concentration in cultures of *Prochlorococcus* sp. PCC 9511. The concentration of the enzyme showed little change upon nitrogen starvation (Fig. 2). On the other hand, the enzyme activity did not change with regard to the samples growing on ammonium (Fig. 3A). Moreover, the levels of intracellular 2-OG showed no significant changes compared to the control culture (Fig. 3B). As it happened in the case of GS regulation in *Prochlorococcus* [17], [24], this is in sharp contrast with the results previously found in *Synechocystis* sp. PCC 6803 [40] and *Phormidium laminosum* [38], where a marked increase of ICDH activity was observed after N starvation. Muro-Pastor *et al* found in *Synechocystis* sp. PCC 6803 a 7-fold increase in the expression of *icd* [40], in agreement with the presence of a NtcA-regulated promoter for the *icd* gene. There might be, however, a certain variability related to differences among isolates, since a glucose-tolerant strain of *Synechocystis* sp. PCC 6803 showed only a 2 to 3-fold increase in *icd* expression after nitrogen depletion [61]. Moreover, in a global expression study on two *Prochlorococcus* strains [62], the authors observed diverging results of N starvation on *icd* expression, depending on the studied strain, since they reported a 2.46-fold increase in the expression of *icd* for the strain MED4 (which is genetically identical to PCC 9511) vs a 3-fold decrease for the strain MIT9313, suggesting a natural variance in response to N starvation.

According to our calculations, the intracellular concentration of 2-OG was ca. 0.05–0.15 nmoles/mg protein under control conditions, in good agreement with the values described for *Synechocystis* sp. PCC 6803 and *Anabaena* sp. PCC 7120 [45], [48], [63]. Therefore, the possible regulatory differences between *Prochlorococcus* and other freshwater model cyanobacteria do not seem to be produced by a general change in the 2-OG concentrations in cells.

Another key nutrient in oligotrophic oceans is phosphorus. As in the case of nitrogen starvation, the activity was very similar in both the control and the starved cultures (not shown). By contrast, GS activity from the same strain experimented a marked decrease under phosphorus starvation [17], [24]. However, the ICDH enzyme concentration decreased (Fig. 2). Recent studies have addressed the effects of P limitation/starvation in *Prochlorococcus* sp. MED4 cultures, showing significant changes, specially in the physiology of P uptake [16] and in the expression of P uptake genes and a P stress regulatory gene [64]. These studies did not report, though, specific effects for ICDH or *icd* expression.

Finally, we studied the effect of iron starvation, another element whose concentration is limiting in many oligotrophic oceans. Lack of iron had a most striking effect, inducing a marked decrease in the number of cells, to the point of making impossible to detect any ICDH activity. However, we found little effect on the ICDH enzyme concentration after 8 hours of starvation (Fig. 2), suggesting a rapid loss of activity. This could be a direct consequence of the oxidative stress induced by iron starvation, as previously reported in other cyanobacteria [65]. Strong iron limitation could provoke the rapid inactivation of ICDH by means of the oxidative stress imposed on *Prochlorococuccus* sp. PCC 9511 cells (see below), while the degradation of the enzyme would require longer times than those studied in this work.

In a previous study [66], we found a general decrease of gene expression in the *Prochlorococcus* sp. strain SS120 under iron starvation, including a decrease of ca. 8 times in the expression of *icd*. In global expression studies in the *Prochlorococcus* strains MED4 and MIT9313 [67] and in *Synechocystis* sp. PCC 6803 [68], a general effect affecting the expression of many genes from different metabolic pathways has been described. Our results fit nicely in this context, suggesting that iron starvation might act by different ways on the metabolism of *Prochlorococcus*, including limitation in the biosynthesis of molecules containing iron, and also more general effects derived from oxidative stress [69], [70].

### Oxidative modification of isocitrate dehydrogenase

The apparent lack of response of ICDH under nitrogen starvation in *Prochlorococcus* might be explained by recent results showing that the redox status of ICDH was clearly affected in *Prochlorococcus* sp. SS120 in those conditions [71]. Since the amino acid residue affected, Cys 463 [71], is conserved in the ICDH of *Prochlorococcus* sp. MED4 (genetically identical to PCC 9511), it likely suggests that this form of regulation might be conserved in this strain, supporting that the lack of response to nitrogen starvation might be directly related to the process of oxidative modification observed in ICDH of *Prochlorococcus* sp. PCC 9511 [25].

**Figure 4:**
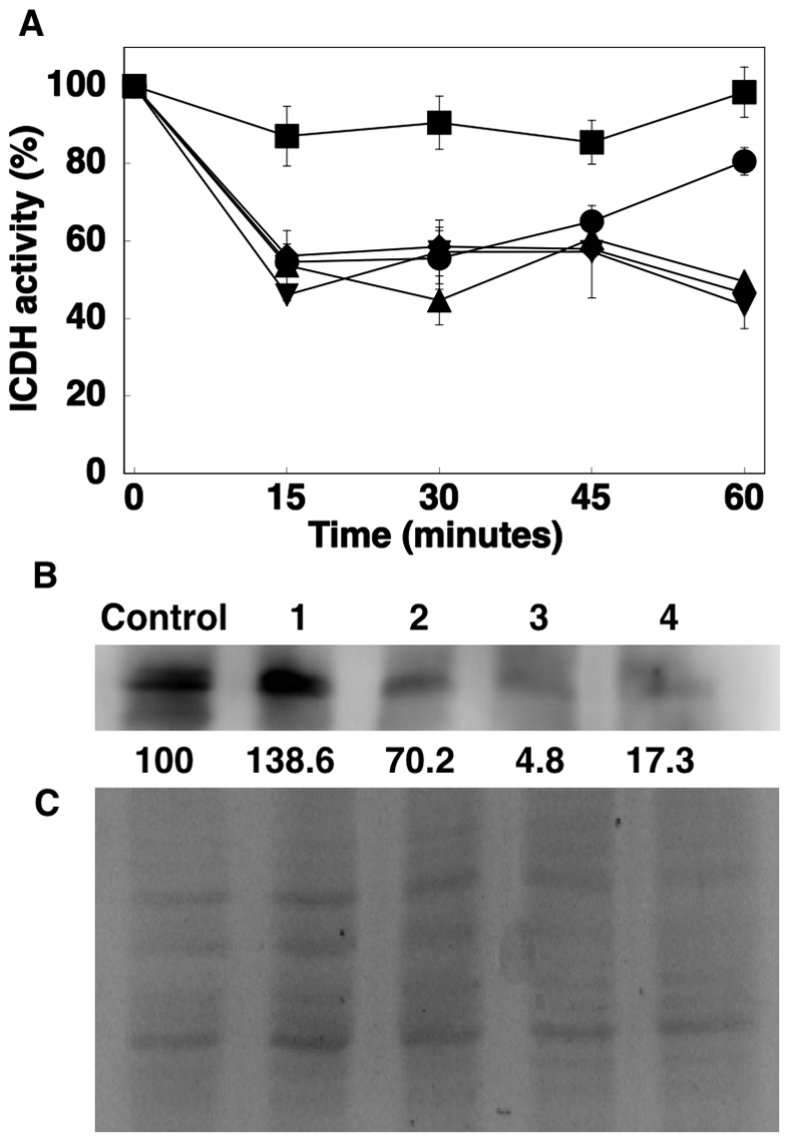
Effect of a metal-catalyzed oxidative system (Fe^3+^/ascorbate) on ICDH activity and concentration. A, Time course of ICDH activity in *Prochlorococcus* sp. PCC 9511 cell extracts with the following additions: ▪, control cells (no addition); •, 0.2 mM FeCl3; ▴, 0.2 mM FeCl3 +1 mM ascorbate; ⧫, 0.2 mM FeCl3 +5 mM ascorbate; ▾, 0.2 mM FeCl3 +10 mM ascorbate. Values are the average of three biological samples, each of them measured in triplicate. Error bars correspond to the standard deviation. B, Western blotting of ICDH in *Prochlorococcus* sp. PCC 9511 cell extracts after 60 min of incubation under the following additions: Control, no addition; 1, 0.2 mM FeCl3; 2, 0.2 mM FeCl3 +1 mM ascorbate; 3, 0.2 mM FeCl3 +5 mM ascorbate; 4, 0.2 mM FeCl3 +10 mM ascorbate. The quantification of bands is shown below the picture, assigning an arbitrary value of 100 to the control conditions. C, Coomasie-stained gel corresponding to a duplicate of that used for the Western blotting shown in B.

Here we further characterized the effect of oxidative modifications on the activity and concentration of ICDH. To this goal, cell extracts of *Prochlorococcus* sp. PCC 9511 were subjected to the effect of a metal-catalyzed oxidative (MCO) system composed by Fe^3+^ and ascorbate (described in detail previously [21], [25]) for 60 min. The results are shown in Fig. 4. Addition of Fe^3+^ alone induced a clear decrease on ICDH activity (Fig. 4A), but not the degradation of the enzyme (Fig. 4B); this effect was enhanced by the addition of ascorbate, which in combination with Fe^3+^ forms a MCO system capable of inactivating GS in enterobacteria [72], cyanobacteria [21], [25] and green algae [73]. Furthermore, this MCO system induced as well the degradation of ICDH after 60 min (Fig. 4B).

### Effect of nitrogen assimilation inhibitors

**Figure 5:**
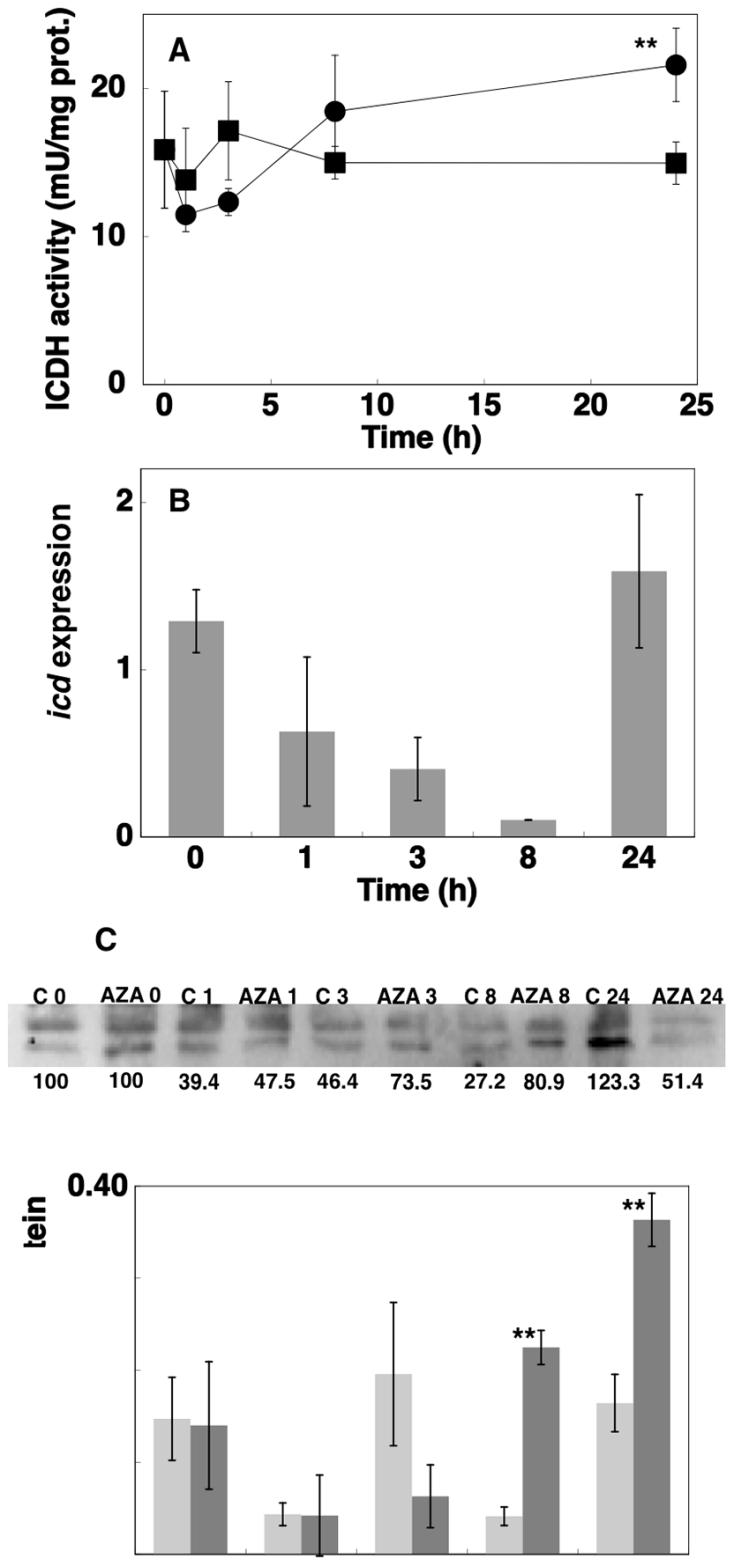
Effects of azaserine addition on ICDH activity, *icd* expression, ICDH enzyme concentration and 2-OG concentration. A, Effect on ICDH activity in *Prochlorococcus* sp. strain PCC 9511 cultures (▪, control cells; •, cells in the presence of 100 µM azaserine). B, Effect on *icd* expression in *Prochlorococcus* sp. PCC 9511 cultures. C, Western blotting from cultures under control conditions or subjected to 100 µM azaserine addition. Lanes are marked with C (control) or AZA (azaserine), followed by sampling time (in hours). Quantitation of bands is shown below the picture, assigning an arbitrary value of 100 to the time 0 of each series (control, azaserine). D, Time course of 2-OG concentration (light grey, control cells; dark grey, azaserine-treated cells). Values are the average of at least three independent biological samples. Error bars correspond to the standard deviation.

In previous studies we observed that some inhibitors of enzymes involved in N metabolism had strong effects on its assimilation [17], [24]. Thus we analyzed ICDH activity and concentration in *Prochlorococcus* sp. PCC 9511 cultures after addition of 100 µM methionine sulfoximine (MSX, an specific inhibitor of GS [74]) and 100 µM azaserine (specific inhibitor of GOGAT [75]). The results are shown in Figs. 2 and 5.

MSX addition did not change significantly ICDH activity (not shown), although the enzyme concentration decreased compared to the control (Fig. 2). However, the effect of azaserine addition provoked a significant increase (*p* = 0.0098, n = 4) in the enzymatic activity (almost 50% after 24 h; Fig. 5A). This result is similar to that found on GS activity in previous studies in *Prochlorococcus* sp. PCC 9511 [17] and *Synechocystis* sp. PCC 6803 [48]. Given such response in enzyme activity, we analyzed the effect of azaserine in more detail, by measuring time-course changes in *icd* expression (Fig. 5B), ICDH enzyme concentration (Fig. 5C) and 2-OG concentration (Fig. 5D). We observed a clear decrease in the concentration of ICDH enzyme 24 h after addition of the inhibitor (Fig. 2 and 5C). However, although *icd* expression decreased sharply until 8 h after azaserine addition, there was a strong recovery observed at 24 h (Fig. 5B). Interestingly, in the SS120 strain, azaserine induced similar changes in the *icd* expression, with an initial repression and a final increase of -ca. 9-fold after 24 h [66]. Maybe this increased *icd* expression is a response to compensate for the loss of ICDH enzyme induced by azaserine. The delay observed for *icd* induction in our results might be explained by the low amount of ribosomes in these slow growing cells, which is reflected in a lag between transcription and protein production that has been reported in *Prochlorocococcus* to be 2–8 h [76].

The promoter of *icd* in *Prochlorococcus* MED4 shows a NtcA binding site where the initial GT nucleotides have been changed to CC [77]. However, a recent study on the transcriptome of *Prochlorococcus* MED4 and MIT9313 presenting genome-wide maps of transcriptional start sites (TSS) for both organisms [78] has not found any TSS which could be related to this NtcA binding site centered at −525 nucleotides with respect to the translational start of *icd*. Furthermore, this work assingns to the *icd* gene a TSS at position −71 but no consensus sequence for NtcA binding can be detected upstream of this TSS. Interestingly, the same study showed that the *icd* gene possesses antisense non-coding RNAs in both strains [78], suggesting a specific control of its expression. This might help to understand the behaviour of *icd* expression upon azaserine addition, observed in *Prochlorococcus* sp. PCC 9511 (Figure 5B) and SS120 ([66]), although no information regarding the possible occurrence of antisense RNAs in the *icd* gene of *Prochlorococcus* sp SS120 is thus far available. Further work is required to explore the importance of antisense RNA regulation in the expression of *icd* in *Prochlorococcus*.

Since blocking GOGAT in *Prochlorococcus* with azaserine should increase the intracellular concentration of 2-OG, we measured its concentration after addition of the inhibitor. The results are shown in Fig. 5D. Azaserine induced a ca. 5.5-fold increase in the concentration of 2-OG after 8 h of the addition; moreover, at 24 h its concentration was still 2.3-fold higher, compared to the controls (in both cases, the results were statistically significant; *p* = 0.005 and 0.001 respectively; n = 3). Hence our results confirm that azaserine addition does provoke a strong increase in the concentration of the key molecule responsible for sensing the C/N balance in cyanobacteria, 2-OG [45], leading to the effects observed on ICDH (this work) and other enzymes, as GS, in *Prochlorococcus* [17], [24].

To discard that the azaserine effect was induced by a direct effect of 2-OG on the ICDH activity, we added 2-OG to the reaction mixture, observing that 6 mM 2-OG provoked a decrease of 30% in ICDH activity, in good agreement with results described in *Synechocystis* sp. PCC 6803 [36]. Hence we can conclude that azaserine provokes a transcriptional effect mediated by the increase in 2-OG concentration (observed at 8 and 24 h; Fig. 5C), and not by a direct effect of 2-OG on the ICDH activity.

Interestingly, azaserine addition induced a strong increase in 2-OG concentration (Fig. 5D), while nitrogen starvation had little effect on the concentration of this metabolite (Fig. 3B). This supports the previously proposed hypothesis [22] that *Prochlorococcus* might be naturally adapted to low nitrogen concentrations in the oceans, which in turn provokes small effects on the C/N regulatory mechanisms. However, azaserine induces a much stronger effect by artificially blocking GOGAT, and thus provoking a sharp increase in 2-OG pools (Fig. 5D), which in turn profoundly affects the C/N metabolism in *Prochlorococcus*, as shown in Fig. 5 and in previous papers from our team [17], [24], [34], [66], [79].

The increases in enzymatic activities observed after azaserine addition in two central enzymes of the C and N metabolism, GS [17] and ICDH (Fig. 5A) indicate that this inhibitor is acting on one of the main players in the metabolic regulation system of *Prochlorococcus*. Furthermore, given that the GS/GOGAT pathway is the only way to metabolize 2-OG in *Prochlorococcus* sp. PCC 9511, it is expectable to observe a stronger effect of azaserine in *Prochlorococcus* than in other model freshwater cyanobacteria with complete TCA. If this hypothesis holds true, azaserine would induce a stronger increase of 2-OG in *Prochlorococcus sp*. PCC 9511 than in *Synechocystis* sp. PCC 6803 [48]. However, the results reported by Mérida and coworkers [48] show that the increase of 2-OG concentration induced by azaserine is actually higher in *Synechocystis* sp. PCC 6803 (7-fold increase vs 2.1-fold increase in *Prochlorococcus* sp. PCC 9511). Interestingly, something similar happens regarding the GS activity upon azaserine addition: it increased 1.67-fold in *Prochlorococcus* sp. PCC 9511 [17] vs 16-fold in *Synechocystis* sp. PCC 6803 [48]. This suggests that for whatever reason (i.e., a lower efficiency of incorporation of azaserine into the *Prochlorococcus* cells), the effect of azaserine is less marked in *Prochlorococcus* sp. PCC 9511 than in *Synechocystis* sp. PCC 6803. Furthermore, previous results from our team suggest that different strains of *Prochlorococcus* might have different sensibilities to the same concentration of azaserine (see table 2 in [17]).

Our results reinforce the idea that 2-OG is also the molecule utilized by *Prochlorococcus* to monitor the C/N balance, as previously proposed [14], [66]. However, the fact that we did not detect significant changes in GS and ICDH under N starvation [17], [24], indicates that possibly *Prochlorococcus* is adapted to live under low N concentration conditions, so the threshold of 2-OG concentration required to trigger the N-limitation response might be higher than in other cyanobacterial genera.

### Effect of darkness and inhibitors of the photosynthetic electron flow

**Figure 6:**
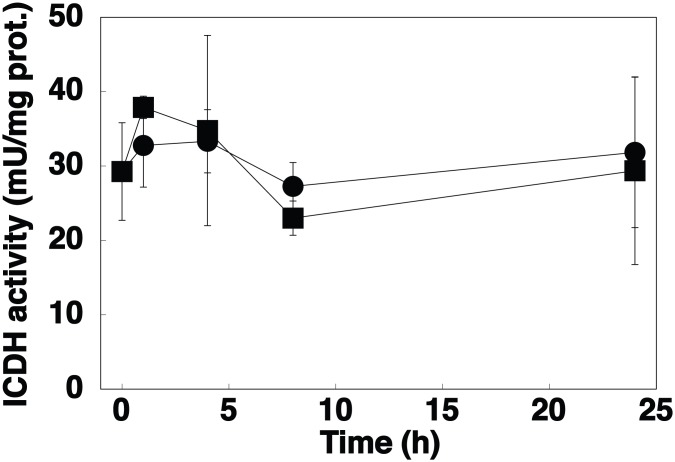
Effect of darkness on the ICDH activity. Time course of ICDH activity in *Prochlorococcus* sp. PCC 9511 cultures. ▪, control cells; •, cells under darkness. Each value shows relative values (cells in the dark vs control cells). Values are the average of three independent biological samples, each of them measured in triplicate. Error bars correspond to the standard deviation.

Light is one of the most important factors in the metabolic regulation of photosynthetic organisms, and is involved in the regulation of gene expression and enzyme activity in cyanobacteria. Thus we studied its effect on the ICDH activity (Fig. 6) and concentration (Fig. 2) in *Prochlorococcus* sp. PCC 9511. Darkness had no effect on the level of ICDH activity (results after 24 h were not significantly different, according to the T-test), but provoked a decrease in the concentration of the enzyme (ca. 60%), in good agreement with the results described for ICDH in *Synechocystis* sp. PCC 6803 [40].

**Figure 7:**
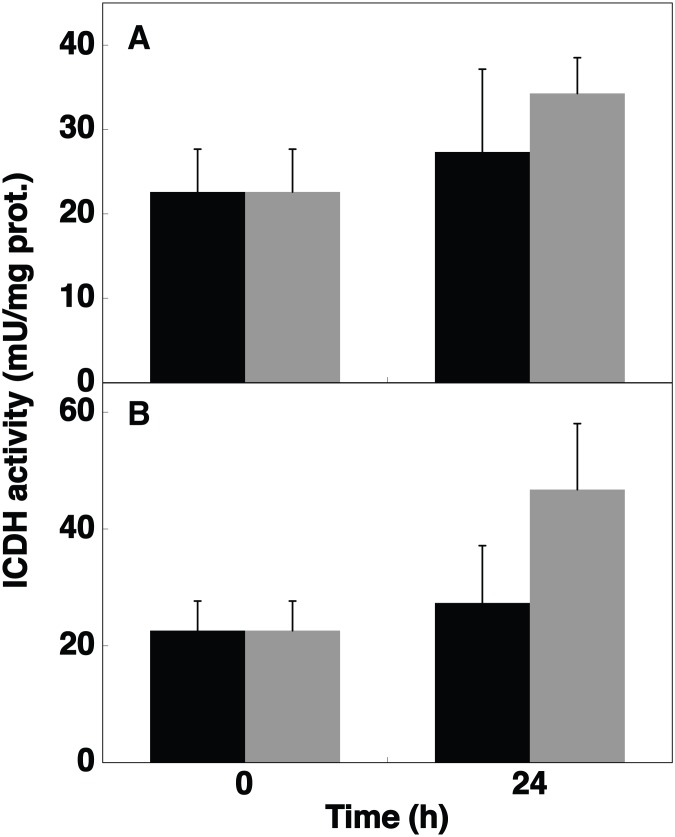
Effect of DCMU and DBMIB on the ICDH activity. A, Changes in ICDH activity in *Prochlorococcus* sp. PCC 9511 cultures after addition of 0.3 µM DCMU. Black, control cells; grey, cells subjected to the DCMU addition. B Changes in ICDH activity in *Prochlorococcus* sp. PCC 9511 cultures after addition of 0.06 µM DBMIB. Black, control cells; grey, cells subjected to the DBMIB addition. Values are the average of three independent biological samples, each of them measured in triplicate. Error bars correspond to the standard deviation.

DCMU and DBMIB are inhibitors of the photosynthetic electron flow, blocking the electron transfer before and after the plastoquinone pool, respectively [80], [81]. DCMU and darkness provoke the oxidation of the plastoquinone pool derived from the decrease in the NADPH intracellular levels, which in turn induces a decrease in the respiratory activity in *Synechocystis* sp. PCC 6803 [82]. When 0.3 µM DCMU was added to *Prochlorococcus* cultures, ICDH activity (Fig. 7A) showed no significant changes after 24 h (*p* = 0.4683), while its concentration (Fig. 2) decreased roughly 50%, compared to the control samples. However, the inhibitor was clearly affecting the cell metabolism at this concentration, as we have shown in previous studies [17].

In the case of DBMIB, the response was completely different: ICDH activity almost doubled with respect to the control sample after 24 h (Fig. 7B; p = 0.081; n = 3), while the enzyme concentration decreased sharply (ca. 80%; Fig. 2). In expression studies carried out in the *Prochlorococcus* strain SS120, *icd* expression showed a striking increase after 8 h (4-fold) to decrease markedly after 24 h [66]. In global expression studies of redox responsive genes in *Synechocystis* sp. PCC 6803, the effects of both DCMU and DBMIB were analysed and no significant change was reported for *icd* [83].

The comparison of the effects of DCMU and DBMIB on the activity and concentration of GS [17] vs ICDH (this work) in *Prochlorococcus* sp. PCC 9511 indicates that their regulatory responses are quite different, although both seem to be affected by the redox status of the plastoquinone pool. This might be related to the fact that GS requires ATP for its catalytic action, while ICDH requires NADP^+^. Since ATP is one of the final products of photosynthesis, blocking the photosynthetic electronic flow would inhibit GS because ATP is missing. On the other hand, NADP^+^ is a substrate for ICDH. Blocking photosynthesis could induce an increase in ICDH activity as an alternate way to generate reducing power under the form of NADPH. Besides, it could be simply a way of ICDH activation because of the increase in one of its substrates (NADP^+^).

### Concluding remarks

The present is the first study analyzing in detail the regulation of ICDH and its relationship with the intracellular pool of 2-OG, under a number of conditions representative of the actual challenges faced by natural *Prochlorococcus* populations. Among the key nutrients in the ocean, the strong effects of iron limitation (reinforced by previous observations from our team [24]) are surprising, as it is commonly considered that *Prochlorococcus* is adapted to low iron concentration. These results suggest that iron is a paramount nutrient for *Prochlorococcus*, and its complete absence can not be compensated even by the adaptive mechanisms developed by this group of cyanobacteria.

Our results suggest that the metabolic regulation of *Prochlorococcus* shares some of the core components with the rest of cyanobacteria, as the sensing of C/N balance by 2-OG. However, there exist some key differences: the different metabolization of 2-OG in marine *Synechococcus* and *Prochlorococcus* vs the rest of cyanobacteria (Fig. 1) might affect in an essential way the standard regulatory mechanisms to control the C/N balance described in freshwater cyanobacteria [46]. In addition, the slight effect of nitrogen starvation on enzymatic activities (this work and previous studies; [17], [24]) might point to a lower threshold for N concentration in the environment, required to start the response through the NtcA regulatory protein. Furthermore, given the large genomic variability observed in *Prochlorococcus*, it would be expectable to observe a certain level of diversity as well in the specific mechanisms controlling the C/N balance in *Prochlorococcus*.

Finally, it is worth noting that the lack of apparent effects on the *icd* expression, ICDH activity and concentration does not preclude the occurrence of regulatory mechanisms involving a change in the physico-chemical properties of the protein, as evidenced by the nitrogen starvation-induced oxidation of the enzyme, reported in previous studies by our team [25], [71] and also shown in this work as effect of a MCO system (Fig. 4).
