# Correction: Physiological Regulation of Isocitrate Dehydrogenase and the Role of 2-Oxoglutarate in *Prochlorococcus* sp. Strain PCC 9511

**DOI:** 10.1371/journal.pone.0110102

**Published:** 2014-09-26

**Authors:** The PLOS ONE Staff

Figure 5 is incorrect. The authors have provided a corrected version here.

**Figure 5:**
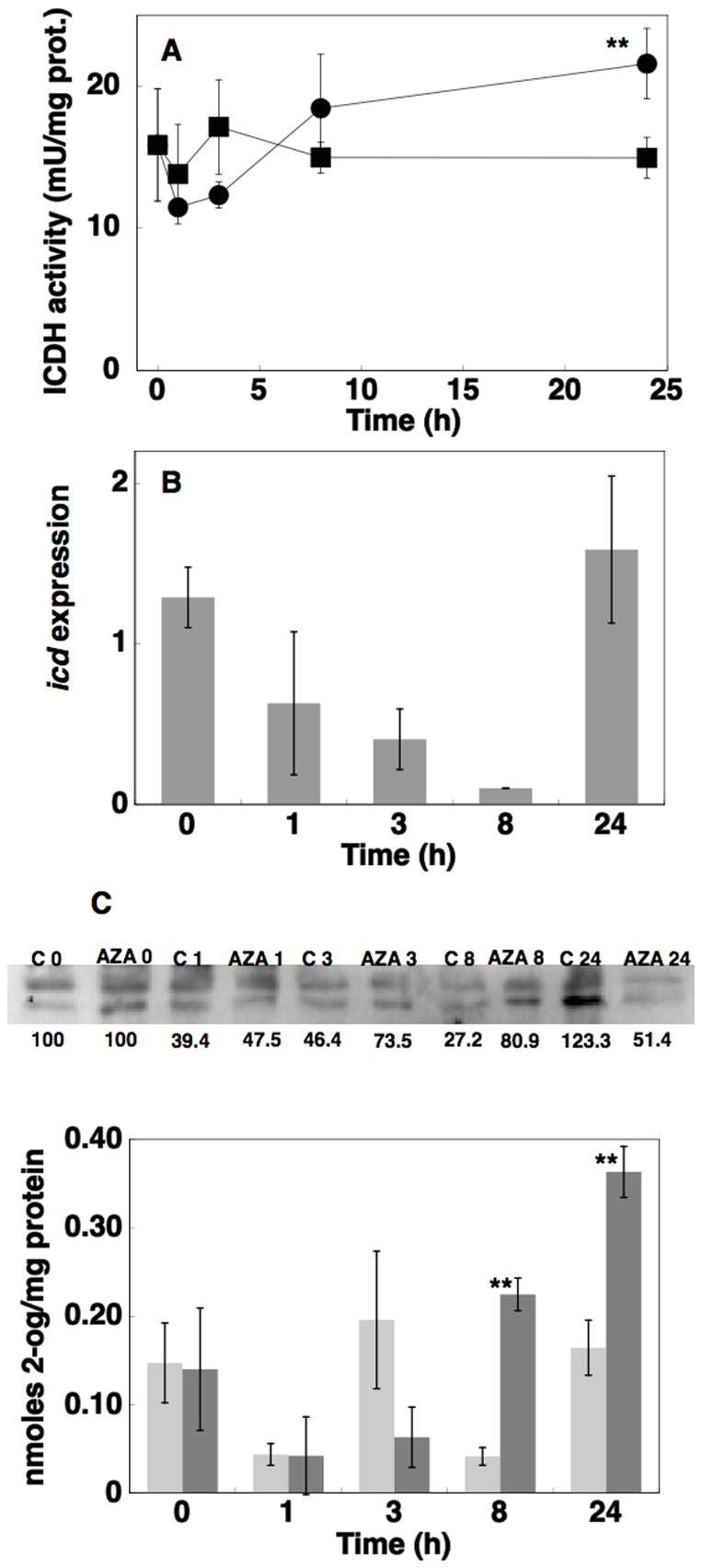
Effects of azaserine addition on ICDH activity, *icd* expression, ICDH enzyme concentration and 2-OG concentration. A, Effect on ICDH activity in *Prochlorococcus* sp. strain PCC 9511 cultures (▪, control cells; •, cells in the presence of 100 µM azaserine). B, Effect on *icd* expression in*Prochlorococcus* sp. PCC 9511 cultures. C, Western blotting from cultures under control conditions or subjected to 100 µM azaserine addition. Lanes are marked with C (control) or AZA (azaserine), followed by sampling time (in hours). Quantitation of bands is shown below the picture, assigning an arbitrary value of 100 to the time 0 of each series (control, azaserine). D, Time course of 2-OG concentration (light grey, control cells; dark grey, azaserine-treated cells). Values are the average of at least three independent biological samples. Error bars correspond to the standard deviation.
